# Diaphragmatic Hernia after Radiofrequency Ablation

**DOI:** 10.3390/diagnostics11020307

**Published:** 2021-02-14

**Authors:** Asahiro Morishita, Joji Tani, Tsutomu Masaki

**Affiliations:** Department of Gastroenterology and Neurology, Kagawa University Faculty of Medicine, 1750-1 Ikenobe Miki-cho, Kita-gun, Kagawa 761-0793, Japan; georget@med.kagawa-u.ac.jp (J.T.); tmasaki@med.kagawa-u.ac.jp (T.M.)

**Keywords:** diaphragmatic hernia, radiofrequency ablation, endoscopic procedure

## Abstract

Diaphragmatic hernia (DH) is a defect, which can be congenital or can develop later in life. Moreover, chromosomal and genetic abnormalities, environmental exposures, and nutritional deficiencies may be related to the development of congenital DH. In contrast, the risk factors of acquired DH include traumas, such as blunt injuries due to traffic accidents and surgical procedures. We report the case of a 71-year-old man admitted to our gastroenterology department for the treatment of esophageal varices. Four days after the endoscopic treatment, the patient vomited severely and reported severe right upper abdominal pain. He was diagnosed with DH, and surgical fixation was performed. The diaphragmatic injury lesion was located on the estimated needle track of percutaneous radiofrequency ablation, which was performed through the thoracic diaphragm with artificial pleural effusion for hepatocellular carcinoma.

Diaphragmatic hernia (DH) is the protrusion of abdominal tissues into the thoracic cavity due to a diaphragmatic defect. While congenital DH is more common with an incidence of approximately 0.8–5 cases/10,000 births [1], acquired DH is rare and occurs following a rupture of the diaphragm due to either a blunt or penetrating trauma. Similarly, a few cases of acquired DH occurring spontaneously or by iatrogenic causes have been reported. Acquired DHs, including iatrogenic DH, can be life-threatening and result in incarceration and strangulation, with a high overall mortality rate [2].

A 71-year-old man was admitted to our gastroenterology department for the treatment of esophageal varices. On admission, endoscopic variceal ligation (EVL) was performed for lower esophageal varices. Four days after EVL, the patient vomited severely and reported severe right upper abdominal pain. 

The patient’s medical history included liver cirrhosis due to hepatitis C virus infection and hepatocellular carcinoma (HCC), which developed at 68 years of age. The patient was treated with radiofrequency ablation (RFA), and no recurrence was observed for 3 years. Similarly, esophageal varices were treated with EVL several times for more than 2 years, and follow-ups were conducted every 6 months.

Laboratory investigation revealed an increase in C-reactive protein levels (4.55 mg/dL) and normal levels of the following parameters; white blood cell count, 4180/μL; neutrophils, 3135/μL; total bilirubin, 1.0 mg/dL; direct bilirubin 0.5 mg/dL; and hemoglobin, 13.8 g/dL. A contrast-enhanced computed tomography revealed a right-sided DH of the colon with numerous stools (Figure 1A,B). Considering the worsening right upper abdominal pain, surgical, surgical repair (fixation) of the hernia was performed. A hole was detected after returning the hernial sac (Figure 2A) and closed with sutures to restore the integrity of the diaphragm and prevent abdominal organs from entering the thoracic cavity. The patient’s clinical condition improved. RFA was performed through the thoracic diaphragm with ultrasound-guided artificial pleural effusion 3 years prior. The lesion of the diaphragmatic injury was located on the estimated needle track of the RFA conducted for HCC in segment 8 (Figure 2B). Following the operation, the patient had liver failure due to deteriorated liver cirrhosis. He slowly recovered and was discharged 57 days postoperatively. 

This is an extremely rare case of a DH of the colon invading the right thorax 3 years following RFA. Air supply during EVL elevated the abdominal pressure, which must have caused perforation of the puncture lesion, i.e., the weakest point of the diaphragm.

Iatrogenic DH caused by RFA is an extremely rare but fatal complication [2]. We reviewed a total of 13 case reports (in PubMed) documenting DH following RFA for HCC. The morbidity rate of diaphragm injury was 0.1% among all adverse events reported after RFA [3]. In addition, Nawa et al. demonstrated that 3 of 10 patients with DH (30%) who underwent RFA died after surgical fixation (average survival after RFA: 20 months) [4]. The tumor location determines the risk of injury to organs adjacent to the liver due to thermal damage caused by RFA [5]. Most previous patients had a history of RFA treatment for HCCs with right dome lesions of segments 7 or 8 and showed a right-sided DH. To avoid diaphragm injury, RFA with artificial pleural ascites targeting tumors near the diaphragmatic surface of the liver should be performed [6]. In this case, DH occurred 36 months after RFA. Remarkably, the area of injury on the diaphragm appeared to be on the needle track to the tumors located in segment 8. It has been reported that poor liver function, hepatic cirrhosis, and other complications causing elevated abdominal pressure (e.g., ascites and ileus) are associated with a higher risk of developing DH [7]. In this patient, EVL may have induced the elevation of abdominal pressure. This case suggests that DH could be a delayed adverse event after RFA and that, if diaphragmatic defect is recognized after RFA, prophylactic surgical repair should be considered.

## Figures and Tables

**Figure 1:**
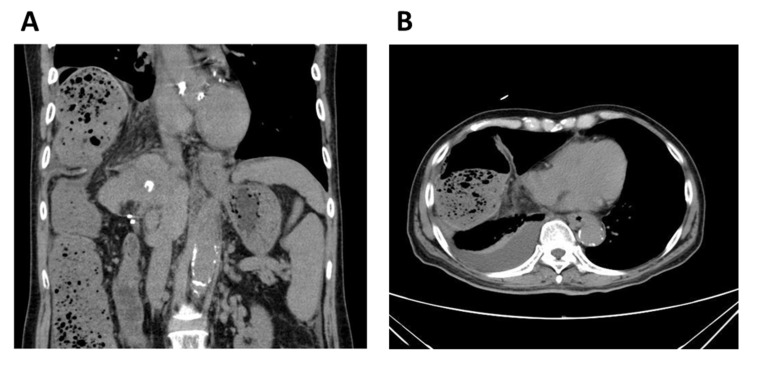
(**A**) Coronal and (**B**) axial views of contrast-enhanced computed tomography reveals a right diaphragmatic hernia of the colon with numerous stools.

**Figure 2:**
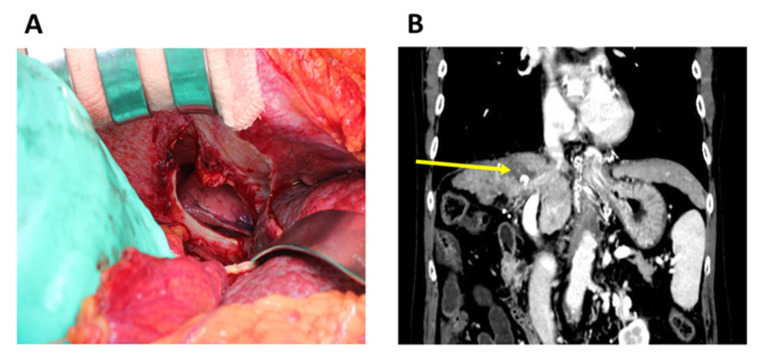
(**A**) Intraoperative images of a hole detected after returning the hernial sac. (**B**) The lesion of the diaphragmatic injury is located on the estimated needle track of the RFA (radiofrequency ablation) conducted for hepatocellular carcinoma (arrow).
